# Supplementary material for: Controlling for baseline telomere length biases estimates of the rate of telomere attrition
Source: R Soc Open Sci. 2019 Oct 30;6(10):190937. doi: 10.1098/rsos.190937 (PMC6837209; doi:10.1098/rsos.190937)
Supplement: Equations S1-S4 [file rsos190937supp1.docx]

**Equations S1-S4. Mathematical proof that mΔLTL is negatively related to LTL measurement error at baseline.**

ΔLTL is estimated from longitudinal datasets in which LTL is measured twice, at baseline (mLTL_b_) and follow up (mLTL_fu_). The measured ΔLTL (mΔLTL) for the *i*th individual is calculated via the following formula:

mΔLTL_i_ = (mLTL_fu.i_ – mLTL_b.i_) (Equation S1)

Thus, a negative value of mΔLTL indicates telomere attrition and a positive value telomere elongation. An individual’s measured LTL can be written as the sum of their true LTL and a measurement error:

mLTL_b.i_ = LTL_b.i_ + error_b.i_ (Equation S2)

mLTL_fu.i_ = LTL_fu.i_ + error_fu.i_ (Equation S3)

Here, error_b.i_ and error_fu.i_ are the errors introduced by measurement for that individual at baseline and follow-up respectively. We assume that error_b.i_ and error_fu.i_ are drawn from independent distributions. Equation 1 can now be expressed in terms of equations S2 and S3:

mΔLTL_i_ = LTL_fu.i_ + error_fu.i_ - (LTL_b.i_ + error_b.i_)

= ΔLTL_i_ + error_fu.i_ - error_b.i_ (Equation S4)

From equation S4 it is evident that there is an inverse relationship between mΔLTL_i_ and error_b.i_. In other words, a larger positive baseline measurement error for an individual results in a more negative mΔLTL, which implies greater measured telomere attrition, for that individual. This is an example of so-called regression to the mean: baseline values are negatively correlated with measures of change because individuals with high mLTL_b_ generally have smaller mLTL_fu_ and vice versa.
