# Supplementary material for: Controlling for baseline telomere length biases estimates of the rate of telomere attrition
Source: R Soc Open Sci. 2019 Oct 30;6(10):190937. doi: 10.1098/rsos.190937 (PMC6837209; doi:10.1098/rsos.190937)
Supplement: Equation S5 [file rsos190937supp2.docx]

**Equation S5. Correction for regression the mean.**

To correct for regression mean we used the equation suggested by Verhulst et al. (1). The change from the baseline measure $X_{1}$ (mLTL_b_) to follow up measure $X_{2}$ (mLTL_fu_) is adjusted for the regression to the mean effect to yield a correct value $D$ as follows:

$D= \rho\left( X_{1}- \bar{X}_{1} \right)-(X_{2}- \bar{X}_{2})$ (Equation S5)

where

$\rho= \frac{2r{S_{1}S}_{2}}{S_{1}^{2}+ S_{2}^{2}}$ (Equation S6)

in which $r$ is the correlation between $X_{1}$and $X_{2}$.

An R function that implements this correction is included in the R script accompanying this paper.

### Reference

1. Verhulst S, Aviv A, Benetos A, Berenson GS, Kark JD. Do leukocyte telomere length dynamics depend on baseline telomere length? An analysis that corrects for “regression to the mean”. Eur J Epidemiol. 2013;28:859–66.
