# Supplementary material for: Controlling for baseline telomere length biases estimates of the rate of telomere attrition
Source: R Soc Open Sci. 2019 Oct 30;6(10):190937. doi: 10.1098/rsos.190937 (PMC6837209; doi:10.1098/rsos.190937)
Supplement: Figure S1 [file rsos190937supp3.docx]

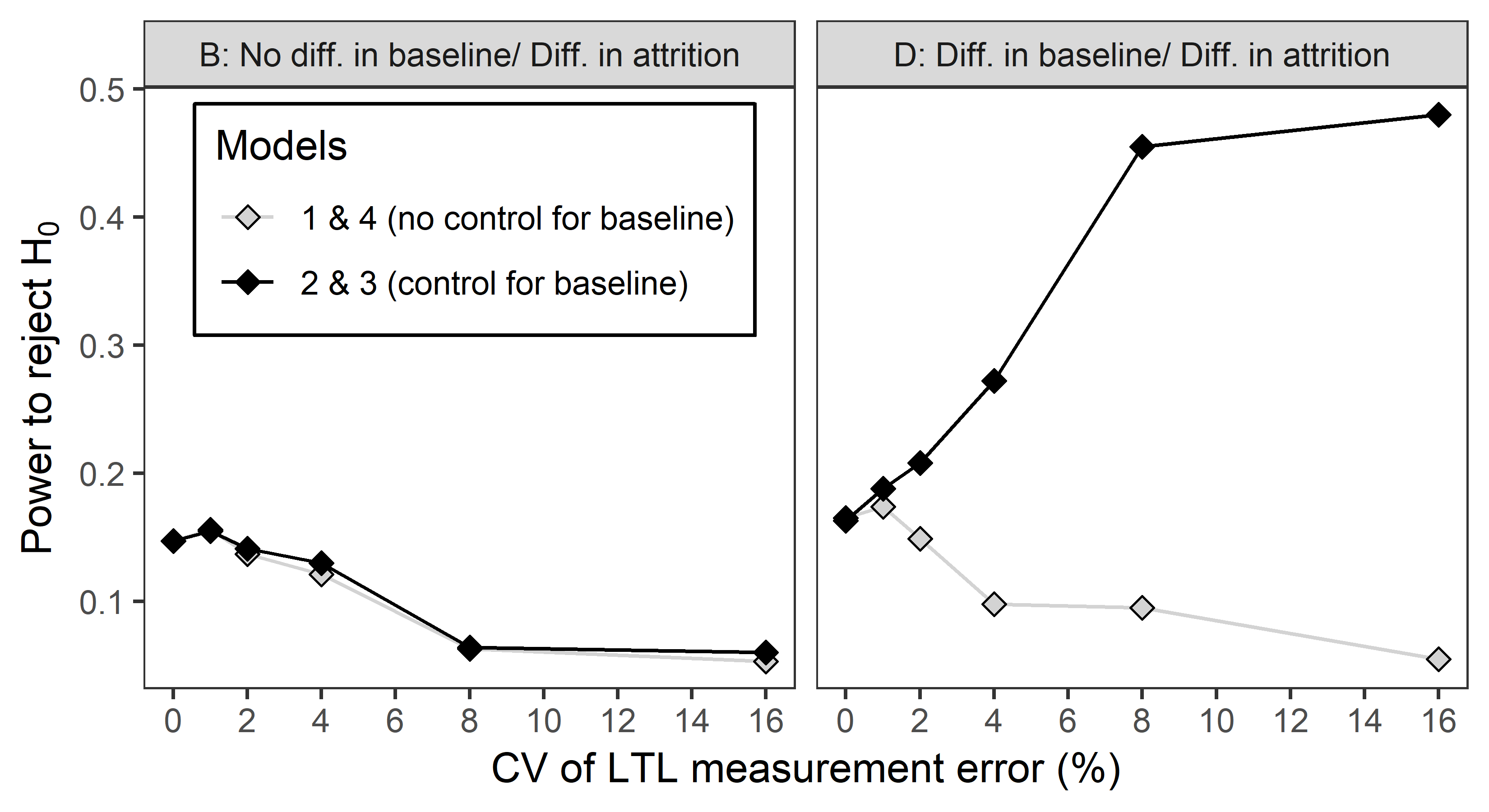


**Figure S1. Controlling for LTL_b_ increases power when there is a difference in LTL_b_.** Power as a function of measurement error (CV) for the four models under consideration. Data points represent the proportion of simulations yielding a p-value below 0.05 in 1000 replicate simulations. The left and right panels show the power in scenarios B and D respectively. The increase in power with increasing CV in scenario D that occurs with models 2 and 3 reflects the bias in parameter estimates for these models shown in Figure 2D. Power is generally low because of the small true effect size assumed in this simulation of only -2 bp.year^-1^. The difference in LTL_b_ between smokers and non-smokers in scenario D was LTL_b_ 141 bp shorter in smokers.
