# Supplementary material for: Controlling for baseline telomere length biases estimates of the rate of telomere attrition
Source: R Soc Open Sci. 2019 Oct 30;6(10):190937. doi: 10.1098/rsos.190937 (PMC6837209; doi:10.1098/rsos.190937)
Supplement: Figure S2 [file rsos190937supp4.docx]

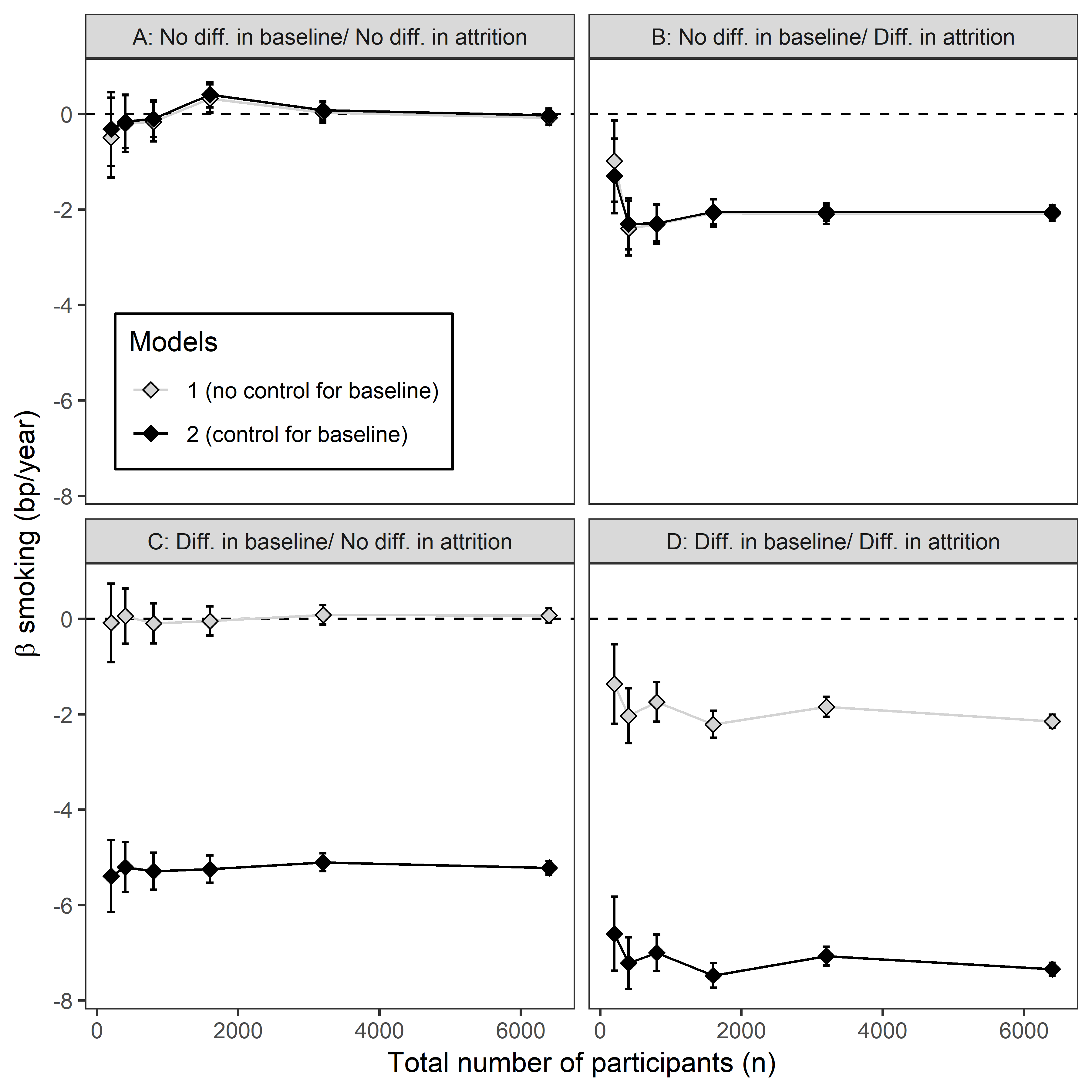


**Figure S2.** **Varying the number of participants in the simulation had no impact on the accuracy of parameter estimates.** Panels show the estimated difference in m∆LTL between smokers and non-smokers as a function of the number of participants in the simulations. The β estimates were obtained by fitting two alternative models to data simulated given four sets of assumptions regarding the true differences between smokers and non-smokers (scenarios A-D in Table 2). The dashed lines indicate no difference in m∆LTL between smokers and non-smokers. Data points are the mean ± 95% confidence intervals obtained from modelling the data from 1000 replicate simulations. The four scenarios were as follows: (A) no difference in LTL_b_ and no difference in ∆LTL; (B) no difference in LTL_b_ but a true difference in ∆LTL; (C) a true difference in LTL_b_ but no difference in ∆LTL; and (D) A true difference in LTL_b_ and a true difference in ∆LTL. The true difference in LTL_b_ between smokers and non-smokers in scenarios C and D was LTL_b_ 141 bp shorter in smokers. The true difference in ∆LTL between smokers and non-smokers in scenarios B and D was ∆LTL -2 bp.year^-1^ greater in smokers. CV was fixed at 8% for this simulation in order to illustrate the impact of varying participant number.
