# Supplementary material for: Controlling for baseline telomere length biases estimates of the rate of telomere attrition
Source: R Soc Open Sci. 2019 Oct 30;6(10):190937. doi: 10.1098/rsos.190937 (PMC6837209; doi:10.1098/rsos.190937)
Supplement: Figure S3 [file rsos190937supp5.docx]

**
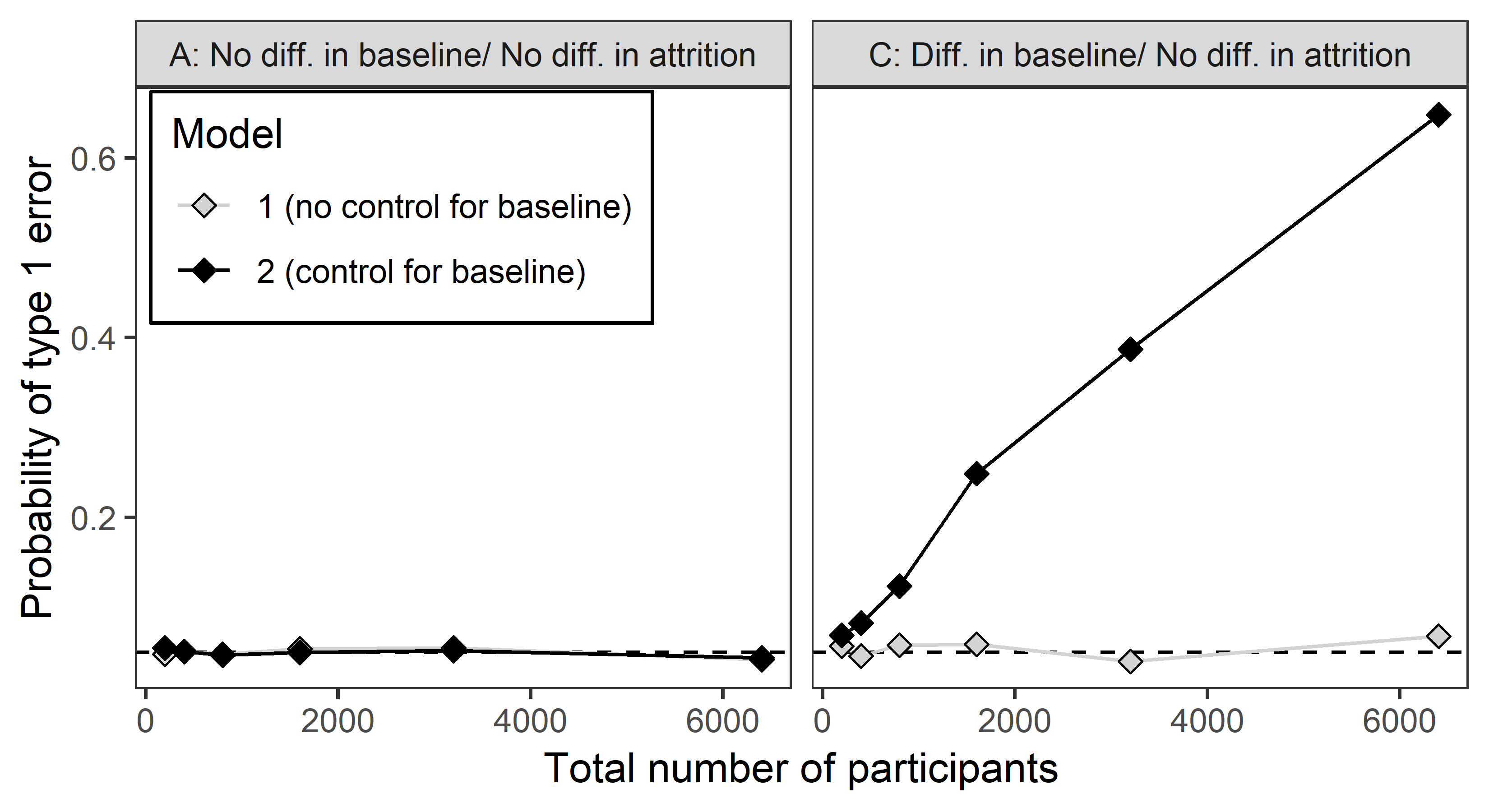
**

**Figure S3. Increasing the number of participants increased the probability of type 1 errors when LTL_b_ was controlled for in scenario C.** Probability of a type 1 error as a function of number of participants for models 1 and 2. Data points represent the proportion of simulations yielding a p-value below 0.05 in 1000 replicate simulations. The left and right panels show the probability of type 1 errors in scenarios A and C respectively. The difference in LTL_b_ between smokers and non-smokers in scenario C was LTL_b_ 141 bp shorter in smokers. CV was fixed at 8% for this simulation in order to illustrate the impact of varying participant number.
