# Supplementary material for: Controlling for baseline telomere length biases estimates of the rate of telomere attrition
Source: R Soc Open Sci. 2019 Oct 30;6(10):190937. doi: 10.1098/rsos.190937 (PMC6837209; doi:10.1098/rsos.190937)
Supplement: Figure S4 [file rsos190937supp6.docx]

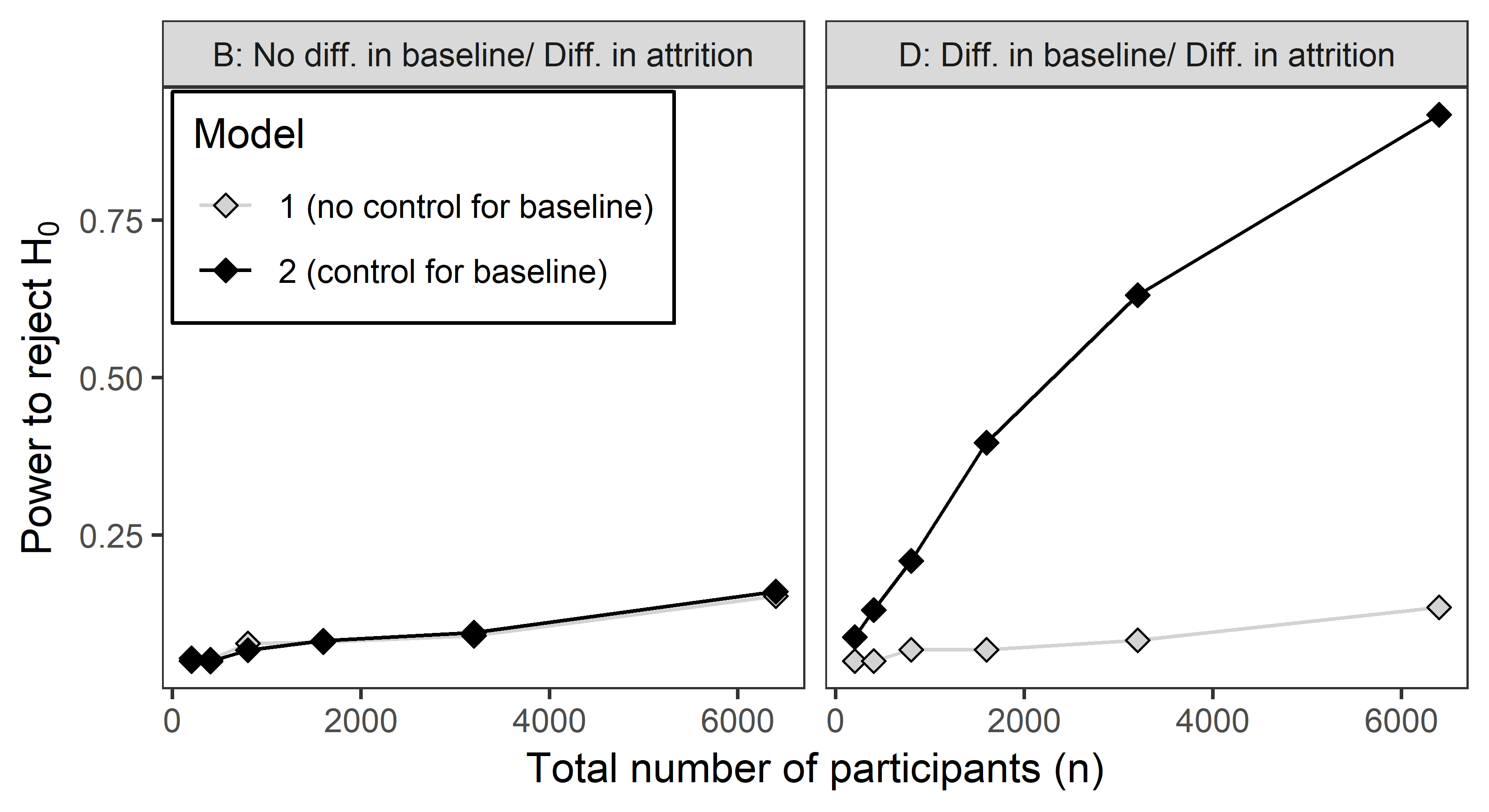


**Figure S4. Increasing the number of participants increased the power in all scenarios and this effect was exaggerated by controlling for LTL_b_ in scenario D.** Power as a function of the total number of participants (n) for models 1 and 2. Data points represent the proportion of simulations yielding a p-value below 0.05 in 1000 replicate simulations. The left and right panels show the power in scenarios B and D respectively. The increase in power with increasing CV seen with model 2 in scenario D reflects the bias in parameter estimates shown in Figure S2D. Power is generally low because of the small true effect size assumed in this simulation (-2 bp.year^-1^). The difference in LTL_b_ between smokers and non-smokers in scenario D was LTL_b_ 141 bp shorter in smokers. CV was fixed at 8% for this simulation in order to illustrate the impact of varying participant number.
