# Supplementary material for: Controlling for baseline telomere length biases estimates of the rate of telomere attrition
Source: R Soc Open Sci. 2019 Oct 30;6(10):190937. doi: 10.1098/rsos.190937 (PMC6837209; doi:10.1098/rsos.190937)
Supplement: Figure S5 [file rsos190937supp7.docx]

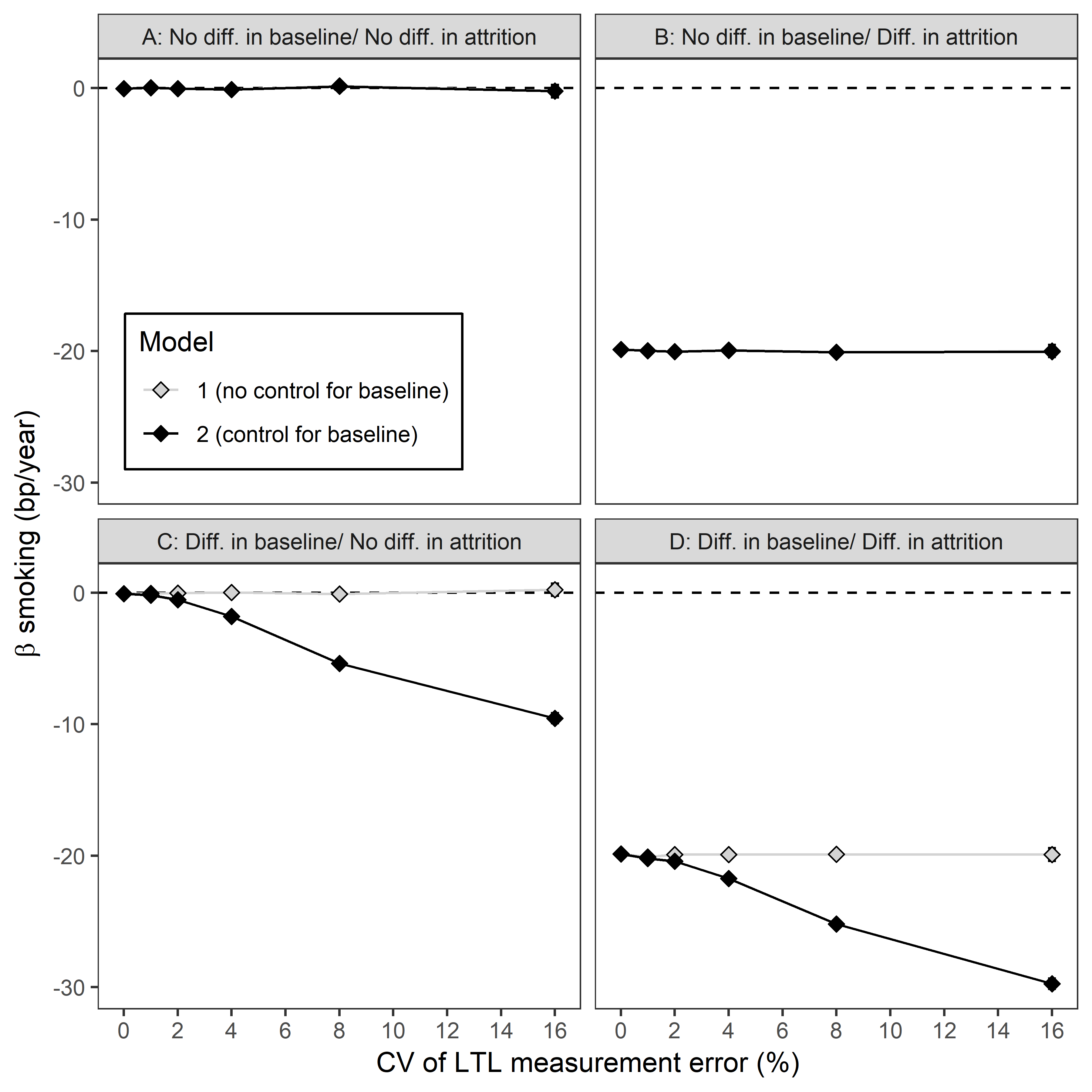


**Figure S5. Increasing the true difference in ΔLTL.year^-1^ between smokers and non-smokers had no further impact on the size of the biases compared to Figure 2.** Panels show the estimated difference in m∆LTL between smokers and non-smokers as a function of measurement error. The β estimates were obtained by fitting two alternative models to data simulated given four sets of assumptions regarding the true differences between smokers and non-smokers. The four scenarios are identical to those given in Table 2, other than that the true difference in ∆LTL between smokers and non-smokers in scenarios B and D was ∆LTL -20 bp.year^-1^ (compared with -2 bp.year^-1^ for the simulation shown in Figure 2). The dashed lines indicate no difference in m∆LTL between smokers and non-smokers. Data points are the mean ± 95% confidence intervals obtained from modelling the data from 1000 replicate simulations.
