# Supplementary material for: Controlling for baseline telomere length biases estimates of the rate of telomere attrition
Source: R Soc Open Sci. 2019 Oct 30;6(10):190937. doi: 10.1098/rsos.190937 (PMC6837209; doi:10.1098/rsos.190937)
Supplement: Figure S7 [file rsos190937supp9.docx]

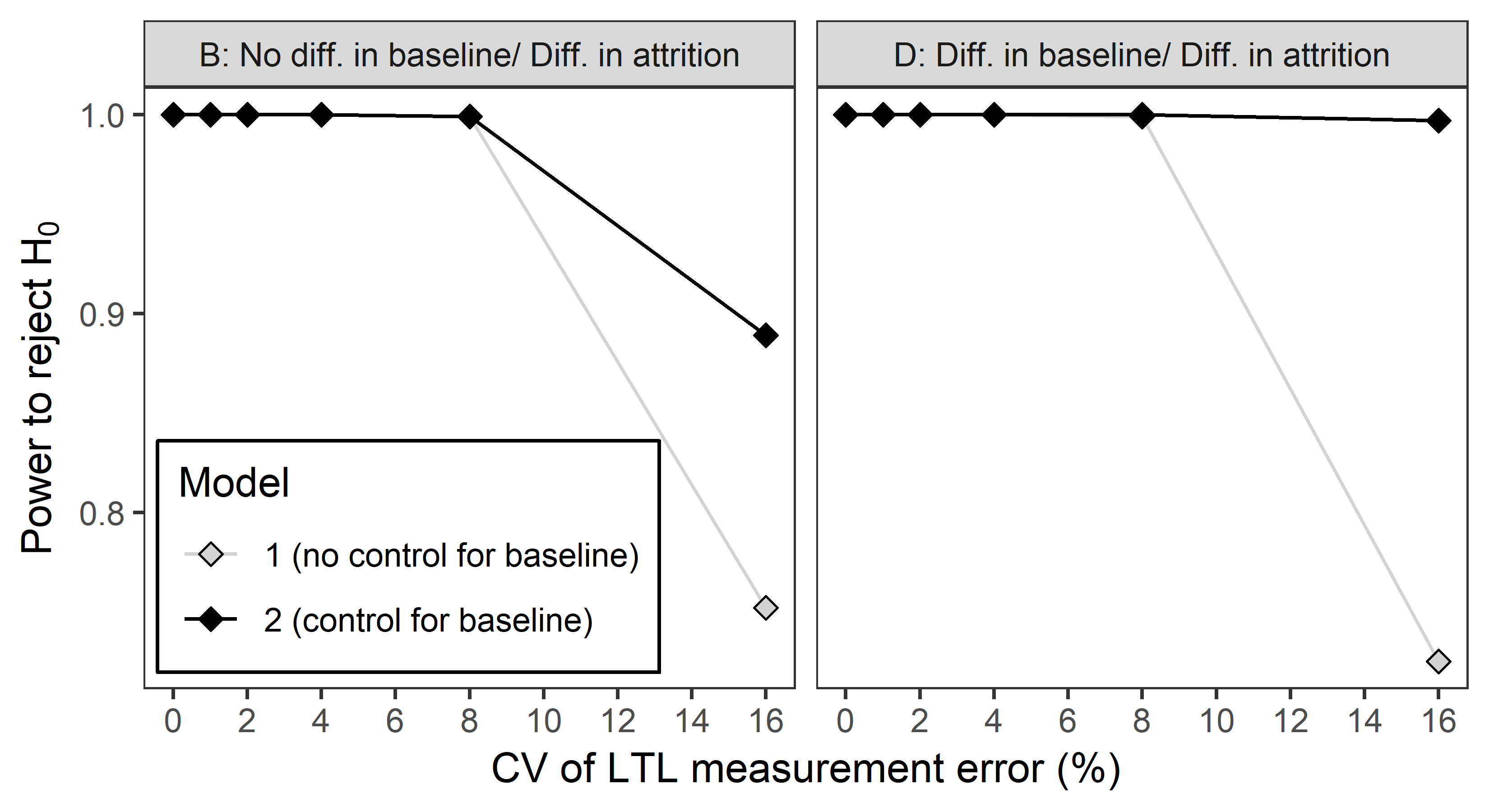


**Figure S7.** **Increasing the true difference in ΔLTL.year^-1^ between smokers and non-smokers increased power compared to Figure S1.** Power as a function of measurement error for models 1 and 2. Data points represent the proportion of simulations yielding a p-value below 0.05 in 1000 replicate simulations. The left and right panels show the power in scenarios B and D respectively. The higher power at a CV of 16% seen with model 2 in scenario D reflects the bias in parameter estimates shown in Figure S5D. Power is generally high because of the large true effect size assumed in this simulation (-20 bp.year^-1^). The difference in LTL_b_ between smokers and non-smokers in scenario D was LTL_b_ 141 bp shorter in smokers.
