# Supplementary material for: Controlling for baseline telomere length biases estimates of the rate of telomere attrition
Source: R Soc Open Sci. 2019 Oct 30;6(10):190937. doi: 10.1098/rsos.190937 (PMC6837209; doi:10.1098/rsos.190937)
Supplement: Figure S8 [file rsos190937supp10.docx]

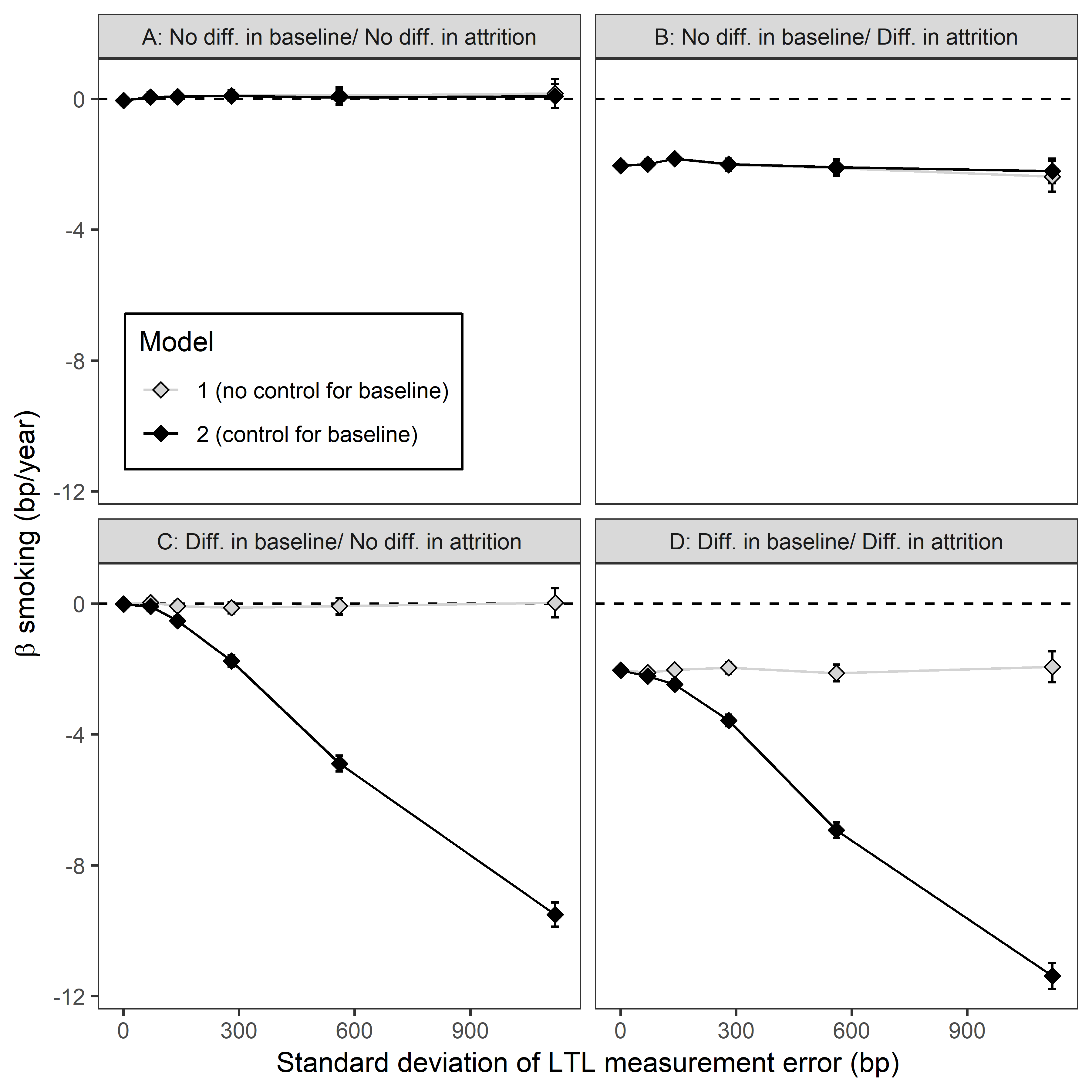


**Figure S8.** **Assuming measurement error to be independent of LTL has no impact on the bias resulting from controlling for LTL_b_ compared to Figure 2.** Panels show the estimated difference in m∆LTL between smokers and non-smokers as a function of measurement error here implemented as a fixed standard deviation (as opposed to as a CV in Figure 2). The β estimates were obtained by fitting two alternative models to data simulated given four sets of assumptions regarding the true differences between smokers and non-smokers. Data points are the mean ± 95% confidence intervals obtained from modelling the data from 1000 replicate simulations. The four scenarios are identical to those given in Table 2 and plotted in Figure 2. The dashed lines indicate no difference in m∆LTL between smokers and non-smokers. The true difference in LTL_b_ between smokers and non-smokers in scenarios C and D was LTL_b_ 141 bp shorter in smokers. The true difference in ∆LTL between smokers and non-smokers in scenarios B and D was ∆LTL -2 bp.year^-1^ greater in smokers.
