# Supplementary material for: Controlling for baseline telomere length biases estimates of the rate of telomere attrition
Source: R Soc Open Sci. 2019 Oct 30;6(10):190937. doi: 10.1098/rsos.190937 (PMC6837209; doi:10.1098/rsos.190937)
Supplement: Figure S9 [file rsos190937supp11.docx]

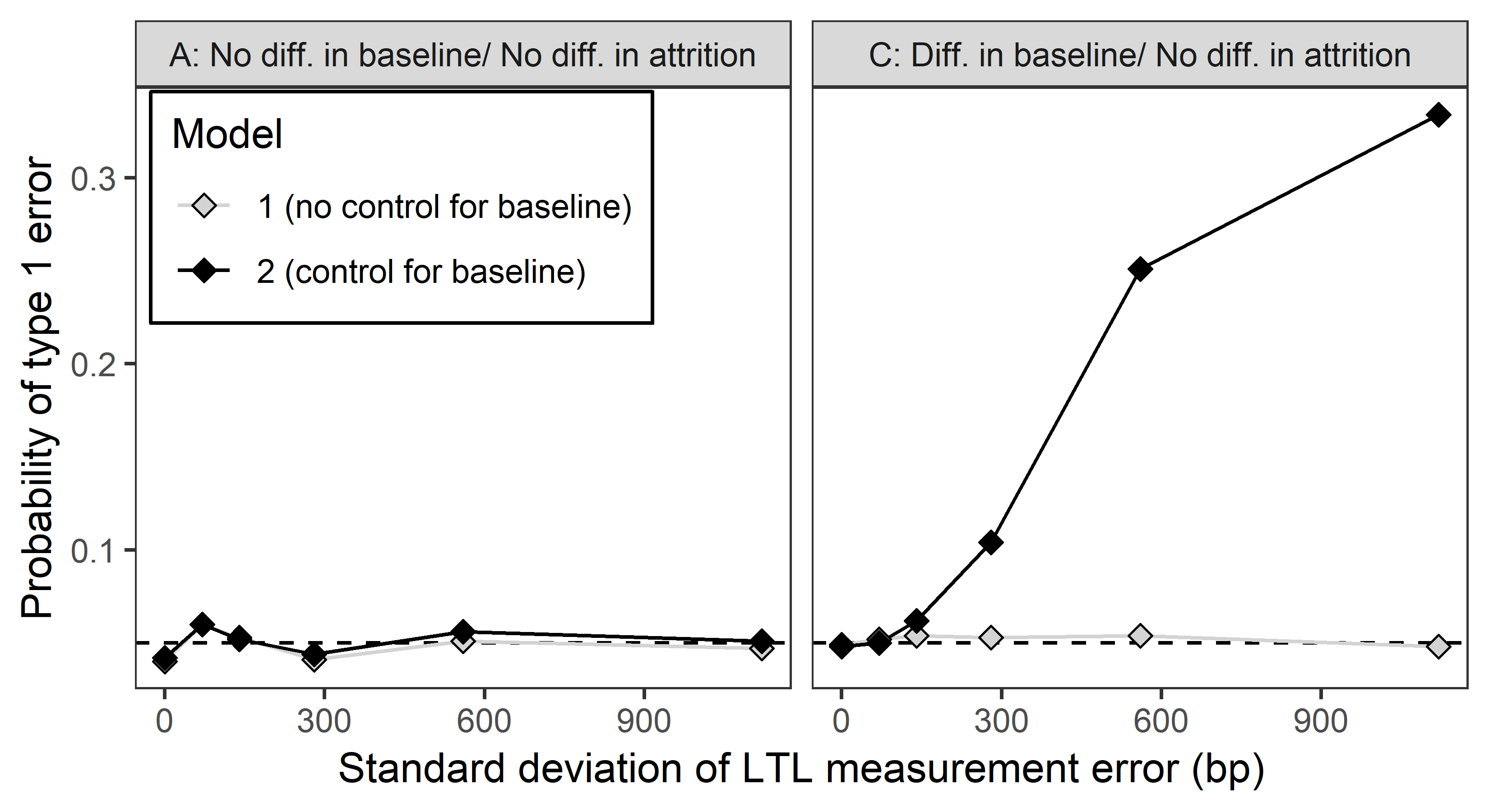


**Figure S9.** **Assuming measurement error to be independent of LTL has no impact on the increased probability of false-positive errors resulting from controlling for LTL_b_ compared to Figure 3.** Probability of a type 1 error as a function of measurement error here implemented as a fixed standard deviation (as opposed to as a CV) for models 1 and 2. Data points represent the proportion of simulations yielding a p-value below 0.05 in 1000 replicate simulations. The left and right panels show the probability of type 1 errors in scenarios A and C respectively. The difference in LTL_b_ between smokers and non-smokers in scenario C was LTL_b_ 141 bp shorter in smokers.
