# Supplementary material for: Controlling for baseline telomere length biases estimates of the rate of telomere attrition
Source: R Soc Open Sci. 2019 Oct 30;6(10):190937. doi: 10.1098/rsos.190937 (PMC6837209; doi:10.1098/rsos.190937)
Supplement: Figure S10 [file rsos190937supp12.docx]

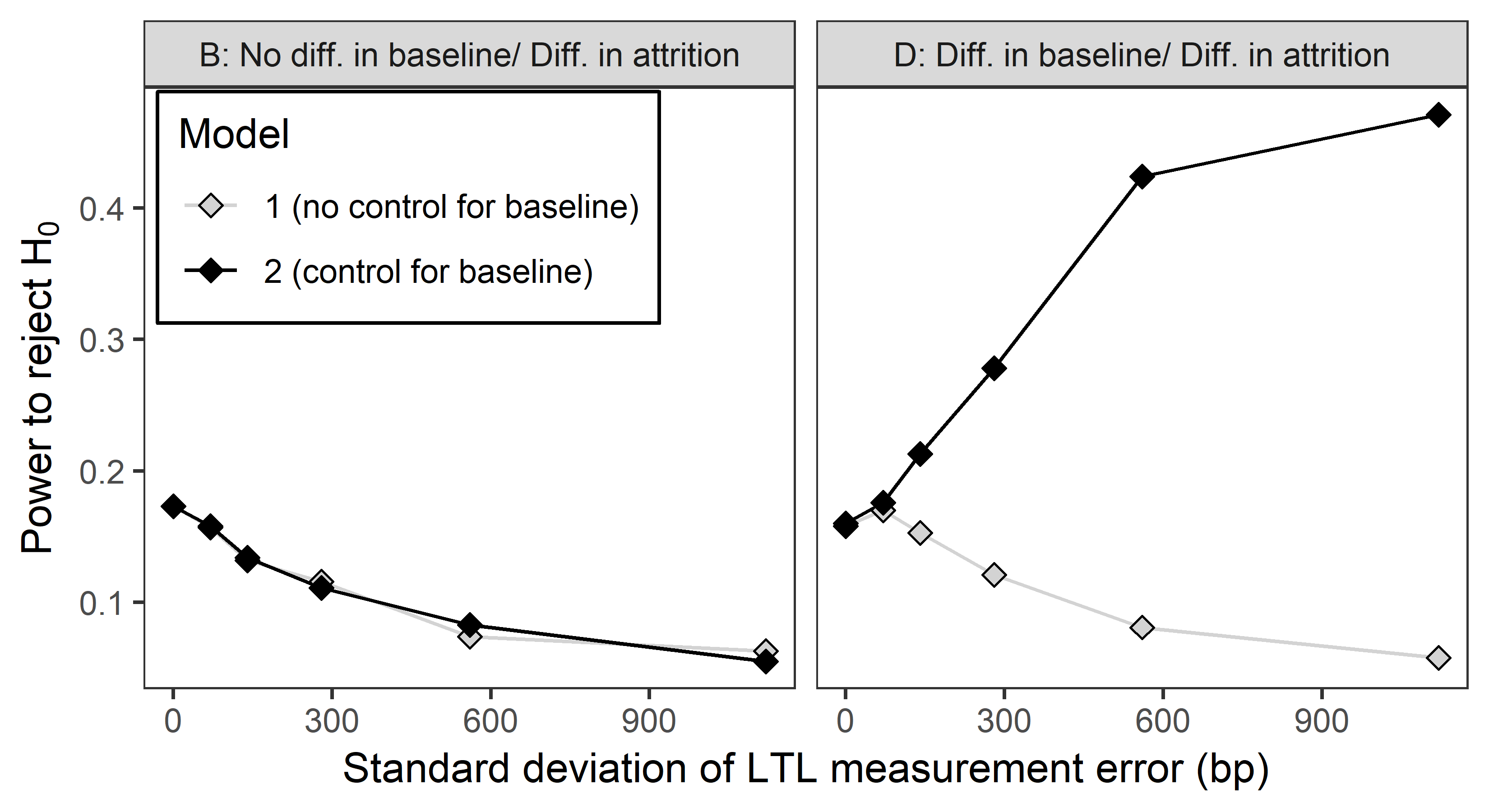


**Figure S10. Assuming measurement error to be independent of LTL has no impact on the increased probability of false-positive errors resulting from controlling for LTL_b_ compared to Figure S1.** Power as a function of measurement error here implemented as a fixed standard deviation (as opposed to a CV) for models 1 and 2. Data points represent the proportion of simulations yielding a p-value below 0.05 in 1000 replicate simulations. The left and right panels show the power in scenarios B and D respectively. The increase in power with increasing measurement error in scenario D that occurs with model 2 reflects the bias in parameter estimates for this model shown in Figure S8D. Power is generally low because of the small true effect size assumed in this simulation of only -2 bp.year^-1^. The difference in LTL_b_ between smokers and non-smokers in scenario D was LTL_b_ 141 bp shorter in smokers.
