# Supplementary material for: Controlling for baseline telomere length biases estimates of the rate of telomere attrition
Source: R Soc Open Sci. 2019 Oct 30;6(10):190937. doi: 10.1098/rsos.190937 (PMC6837209; doi:10.1098/rsos.190937)
Supplement: Figure S12 [file rsos190937supp14.docx]

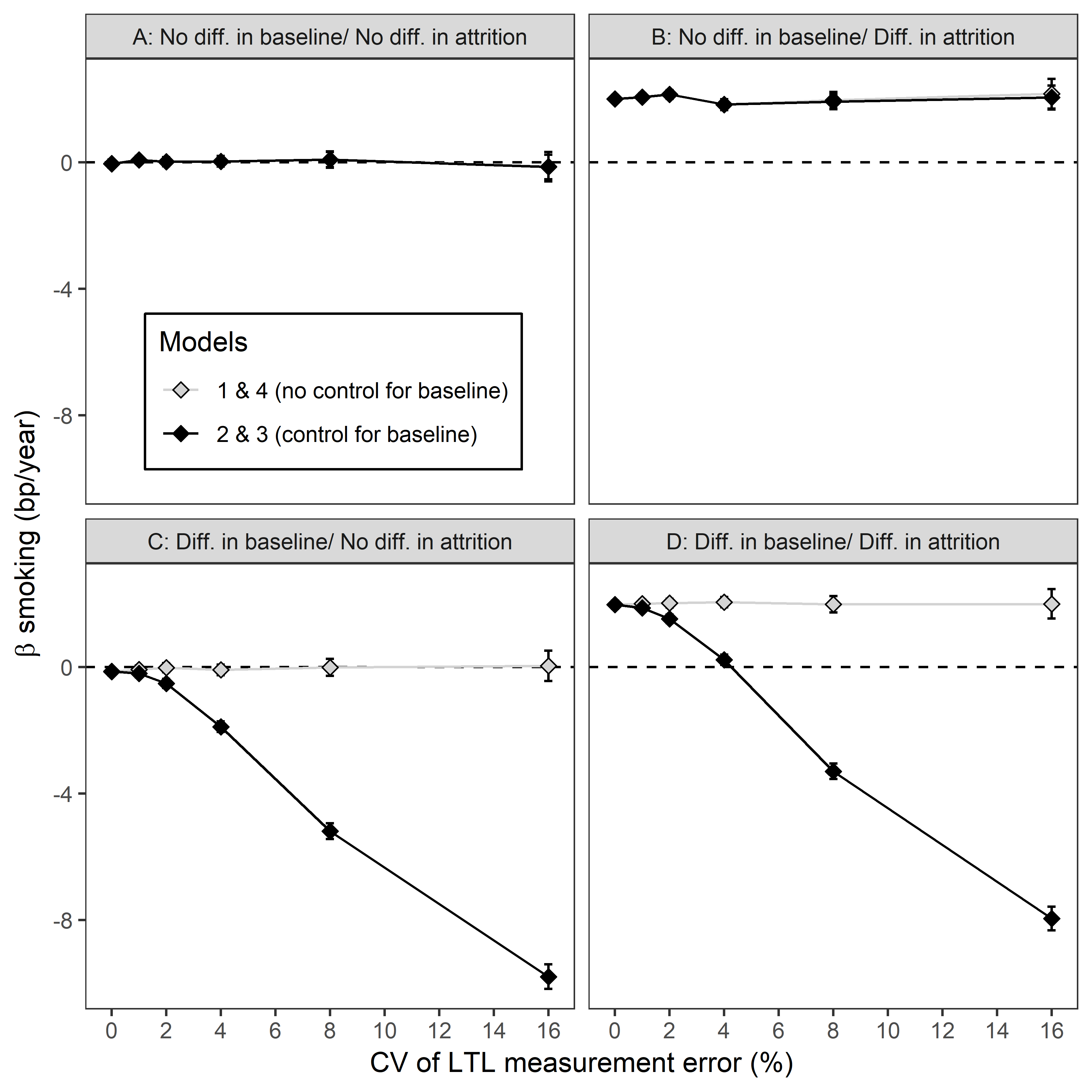


**Figure S12.** **When there are systematic differences in LTL_b_ controlling for LTL_b_ can either eliminate or even reverse estimates of the true difference in ∆LTL between smokers and non-smokers (see panel D).** Panels show the estimated difference in m∆LTL between smokers and non-smokers as a function of measurement error (CV). The β estimates were obtained by fitting four alternative models to data simulated given four sets of assumptions regarding the true differences between smokers and non-smokers. The four senarios are as in Table 2, with the exception that the true difference in ∆LTL in scenarios B and D was reversed, such that ∆LTL was 2 bp.year^-1^ less in smokers (i.e. β smoking = 2 bp.year^-1^). The dashed lines indicate no difference in m∆LTL between smokers and non-smokers. Data points are the mean ± 95% confidence intervals obtained from modelling the data from 1000 replicate simulations. Panel D shows a scenario in which the true difference in ∆LTL is eliminated (β is estimated as ~0) when CV is equal to 4% and reversed when CV is greater than 4% when models 2 and 3 are used.
