# Supplementary material for: Controlling for baseline telomere length biases estimates of the rate of telomere attrition
Source: R Soc Open Sci. 2019 Oct 30;6(10):190937. doi: 10.1098/rsos.190937 (PMC6837209; doi:10.1098/rsos.190937)
Supplement: Figure S13 [file rsos190937supp15.docx]

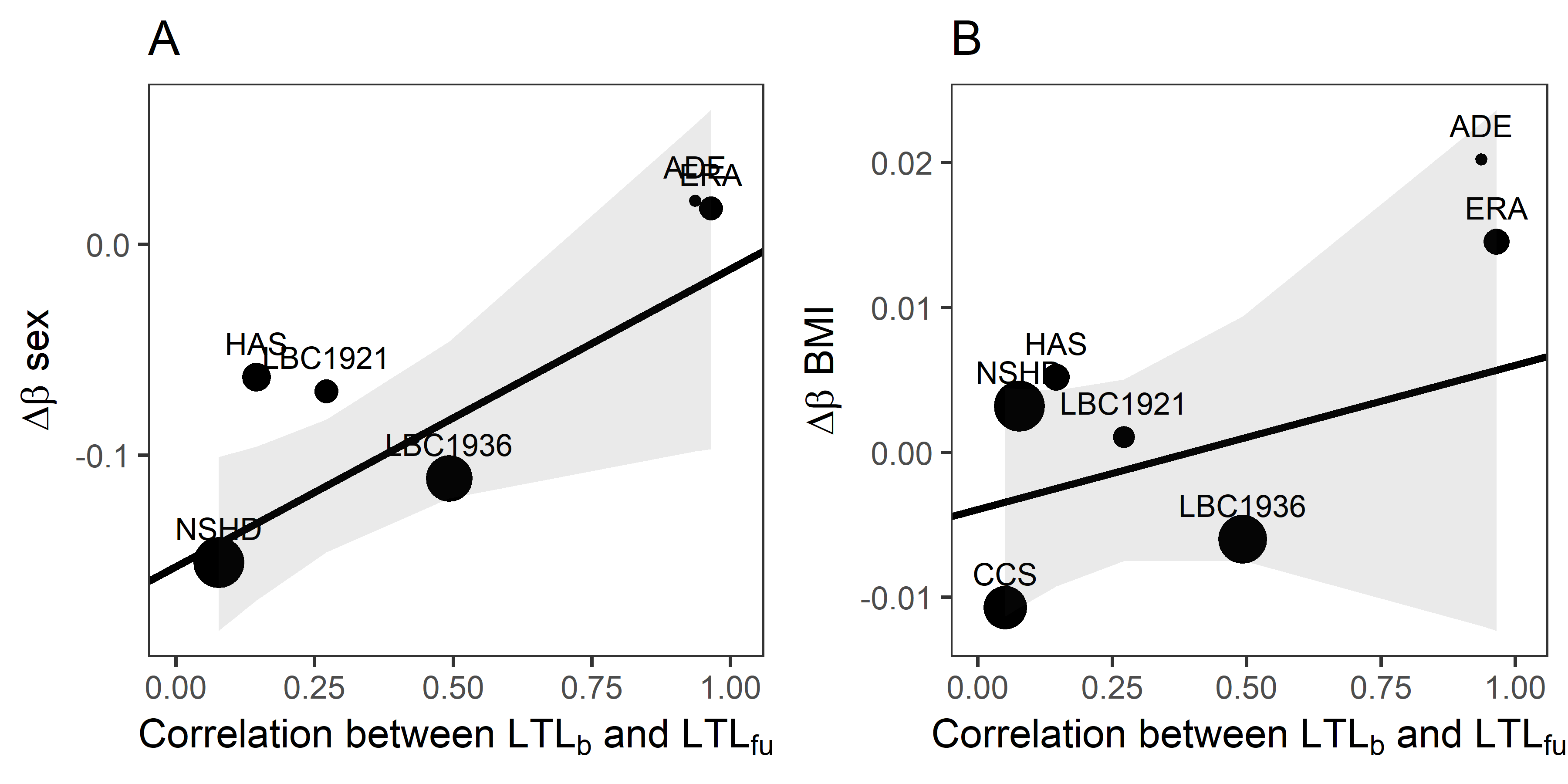


**Figure S13. Measurement error predicts biases for sex and BMI.** The combined dataset available for analysing effects of sex and BMI included data from 3,313 adults, comprising 2,077 males and 1,236 females (see Table S1). As observed for smoking, there is a positive relationship between the LTL_b_-LTL_fu_ correlation coefficient (a proxy for measurement error) and Δβ (the difference between the estimates derived from models 1 and 2) for both sex and BMI**.** A: Scatterplot showing the relationship between the correlation between LTL_b_ and LTL_fu_ and the difference between the β coefficients for sex derived from models 1 and 2 (weighted linear regression for sex: **β±se = 0.14±0.06, t = 2.43, p = 0.0722).** B: As panel A, but the β coefficients are for BMI (weighted linear regression for BMI **β±se = 0.01±0.01, t = 0.82, p = 0.4480).** In both panels, **the solid black line was derived from a linear regression in which the points were weighted by the number of participants in each cohort and the grey ribbon shows the 95% confidence interval for this line.**
