# Supplementary material for: Controlling for baseline telomere length biases estimates of the rate of telomere attrition
Source: R Soc Open Sci. 2019 Oct 30;6(10):190937. doi: 10.1098/rsos.190937 (PMC6837209; doi:10.1098/rsos.190937)
Supplement: Table S1 [file rsos190937supp16.docx]

Table S1. Summary of the datasets analysed for sex and BMI.

| **Cohort^a^** | **Number of participants** | | **Correlation between LTL_b_ and LTL_fu_** | **Differences between sexes in telomere length/attrition^b^**  **(standardised β [s.e.])** | | | **Associations between BMI and telomere length/attrition^c^**  **(standardised β [s.e.])** | | |
| --- | --- | --- | --- | --- | --- | --- | --- | --- | --- |
|  |  | |  | **LTL_b_** | **∆LTL.year^-1^** | | **LTL_b_** | **∆LTL.year^-1^** | |
|  | **Male** | **Female** |  |  | **Model 1** | **Model 2** |  | **Model 1** | **Model 2** |
| ADE | 33 | 35 | 0.94 | -0.55 [0.23] | -0.18 [0.24] | -0.16 [0.26] | -0.22 [0.12] | 0.14 [0.12] | 0.16 [0.13] |
| CCS | 756 | 0 | 0.05 | NA^d^ | NA^d^ | NA^d^ | 0.01 [0.04] | 0.023 [0.04] | 0.012 [0.02] |
| ERA | 108 | 54 | 0.96 | -0.065 [0.17] | -0.01 [0.17] | 0.01 [0.16] | -0.06 [0.08] | -0.05 [0.08] | -0.04 [0.08] |
| HAS | 158 | 95 | 0.15 | 0.09 [0.13] | 0.13 [0.13] | 0.06 [0.09] | -0.01 [0.06] | 0.06 [0.06] | 0.07 [0.04] |
| LBC1921 | 78 | 81 | 0.27 | 0.37 [0.16] | -0.12 [0.16] | -0.19 [0.16] | -0.01 [0.08] | -0.01 [0.10] | -0.01 [0.08] |
| LBC1936 | 444 | 414 | 0.49 | 0.38 [0.07] | 0.21 [0.07] | 0.09 [0.07] | 0.02 [0.03] | 0.00 [0.03] | -0.00 [0.03] |
| NSHD | 500 | 557 | 0.08 | 0.19 [0.06] | 0.25 [0.06] | 0.10 [0.04] | -0.00 [0.03] | -0.01 [0.03] | -0.01 [0.02] |

Notes: ^a^Acronyms for cohorts as defined in Table 3 of the main text. ^b^For sex, positive standardised βs indicate that males have longer LTL_b_ and greater mΔLTL.year^-1^. ^c^For BMI, we used the mean of BMI at baseline and follow-up where this was available; and otherwise either BMI at baseline or BMI at follow-up, whichever was available. For BMI, positive standardised βs indicate that participants with higher BMI have longer LTL_b_ and greater mΔLTL.year^-1^. ^d^We were unable to analyse the effect of sex for the Caerphilly Cohort Study (CCS), since this cohort was restricted to male participants.
